# The Influence of Lyophilization Pretreatment and Whey Content on Whey and Gelatin-Based Hydrogels

**DOI:** 10.3390/gels10040229

**Published:** 2024-03-28

**Authors:** Pompilia Mioara Lopes, Radu Fechete, Felicia Minteuan, Liviu Mare, Dumitrița Moldovan, Marioara Moldovan, Stanca Cuc, Codruța Liana Saroși, Violeta Popescu

**Affiliations:** 1 Physics and Chemistry Department, Technical University of Cluj-Napoca, 28 Memorandumului Street, 400114 Cluj-Napoca, Romania; mioara.lopes@im.utcluj.ro (P.M.L.); rfechete@phys.utcluj.ro (R.F.); minteuanfelicia@yahoo.com (F.M.); mareliviumarius@gmail.com (L.M.); dumitrita.moldovan@phys.utcluj.ro (D.M.); 2 “SAMUS” Special Vocational School, 17 Ialomiței Street, 400574 Cluj-Napoca, Romania; 3 Polymeric Composite Laboratory, Institute of Chemistry “Raluca Ripan”, Babeș-Bolyai University, 30 Fântânele Street, 400294 Cluj-Napoca, Romania; marioara.moldovan@ubbcluj.ro (M.M.); stanca.boboia@ubbcluj.ro (S.C.); codruta.sarosi@ubbcluj.ro (C.L.S.)

**Keywords:** whey and gelatin, hydrogel, crosslinking, lyophilization, mechanical properties

## Abstract

Whey and gelatin, natural polymers within the protein category, find widespread use in hydrogel formulations applied across the food, medical, and pharmaceutical industries. This study presents new characteristics of hydrogels based on whey, gelatin, and copper sulfate as a consequence of the additional steps in the preparation method, specifically refrigeration and freezing storage followed by lyophilization. The water state in hydrogels prior to lyophilization impacts the morphological appearance, with refrigerated hydrogels exhibiting a more regular and dense pore distribution, as shown by the Scanning Electron Microscopy (SEM) images. This observation aligns with the higher mobility of polymer chains indicated by *T*_2_ distributions in ^1^H nuclear magnetic resonance (RMN) relaxometry measurements. Changes in the intensity and amide-specific wavenumbers of the FTIR spectra of whey and gelatin proteins are evident in the Fourier Transformed Infrared (FTIR) spectra of crosslinked and frozen hydrogels before lyophilization. Moreover, the reinforcing effect in the hydrogel matrix, noted in mechanical tests, is attributed to increased polymer chain content and copper sulfate crosslinking.

## 1. Introduction

As a distinct class of polymeric materials, hydrogels are characterized by their hydrophilic functional groups dispersed along the polymer chains, granting them the remarkable capacity to absorb and retain large volumes of water. Hydrogels exhibit specific responses to a range of stimuli, including variations in pH, ionic strength, and exposure to light, water, and other substances [1,2,3]. This capability enables researchers to adjust the composition of hydrogels, tailoring them to provide the desired responses under specific environmental conditions. Alongside carbohydrates, proteins are largely employed in the formulation of hydrogels, owing to their easily tailored characteristics for various applications such as controlled drug transport and release systems, materials with absorbent and antimicrobial properties in pharmacy, medicine, the food industry, and environmental protection [4,5,6,7].

Proteins, especially gelatin and whey, are favored in hydrogel formulations for their excellent gel-forming capabilities. However, their lack of stability in moist environments necessitates the use of crosslinking agents to immobilize hydrophilic segments on protein chains, enhancing their stability [8,9,10]. Crosslinking with metal salts [11,12,13,14] determines the improvement in hydrogel stability in aqueous environments. Using copper compounds for crosslinking has emerged as a preferred method due to the copper’s compatibility and cost-effectiveness. Copper not only serves as an effective crosslinker but also imparts antimicrobial properties to hydrogels [11,12,13,14].

Copper salts are recognized for their overall safety in agricultural [15], aquacultural [16], food packaging [17,18], medical [19], and pharmaceutical contexts [20]. They offer a controllable degree of crosslinking through concentration adjustments, allowing for tailored mechanical properties and degradation rates in moist environments [21,22].

In the production of hydrogels, water plays a determining role as a plasticizer but is also the primary factor that can degrade their quality. Hence, it is crucial to employ suitable drying methods to eliminate moisture from these materials. Freeze-drying is recognized for its ability to increase the porosity of the hydrogel, preserving the hydrogel’s integrity and shape while preventing microbial growth due to water removal [23]. Lyophilization, or freeze-drying, involves a process for extracting moisture from a product at temperatures lower than typical freezing points by causing the ice to undergo sublimation and leading to the formation of cryogels lyophilized hydrogels [4]. These can be incorporated into various food items, such as desserts, ice creams, creams, and sausages as flavor carrier oleogels and then gradually release the components [5,24]. In addition to these aspects, cryogels can play a crucial role in food preservation, offering innovative packaging and storage methods and maintaining the freshness and quality of food products for longer periods. In contrast to conventional methods like warm air drying or ambient temperature drying, freeze-drying completely prevents the potential for natural microbial growth during the drying of organic materials [25], ensuring product stability. On the other hand, there is minimal shrinkage or deformation during the process, which also helps to enhance the absorbent properties [26] due to the large specific surface and high porosity [4]. Manzocco et al. reported pore sizes on the order of micrometers for whey aerogels prepared using the supercritical drying (<1 µm) and freeze-drying (2–5 µm) methods [24], demonstrating that the drying process can influence the properties of whey-based hydrogels.

While the freeze-drying method may incur higher costs, many research papers report that it offers distinct advantages during the preparation of gelatin and whey-based hydrogels, such as forming a self-supporting protein matrix characterized by enhancing their capacity for shape retention and extending the shelf life of printed scaffolds [26], improved mechanical and swelling properties of a whey aerogel for loading lipophilic components [24], over 96% porosity and up to 29.26 g/g oil absorption capacity in synergy with the crosslinking effect of tannic acid [5].

In this study, we introduce an innovative approach to the fabrication of freeze-dried hydrogels composed of whey, gelatin, glycerol, and copper sulfate, utilizing the hydrogel synthesis methods previously outlined [21,27,28]. For the first time, we explore the effect of varying whey concentrations and examine the impact of two distinct pretreatment storage conditions—refrigeration and freezing—on the morphological, structural, and mechanical properties of the hydrogels. This investigation sheds light on how these preparatory steps influence the final characteristics of hydrogels, offering new insights into optimizing their performance for specific applications.

## 2. Results and Discussion

### 2.1. Morphology Characterization by SEM

**Figure 1:**
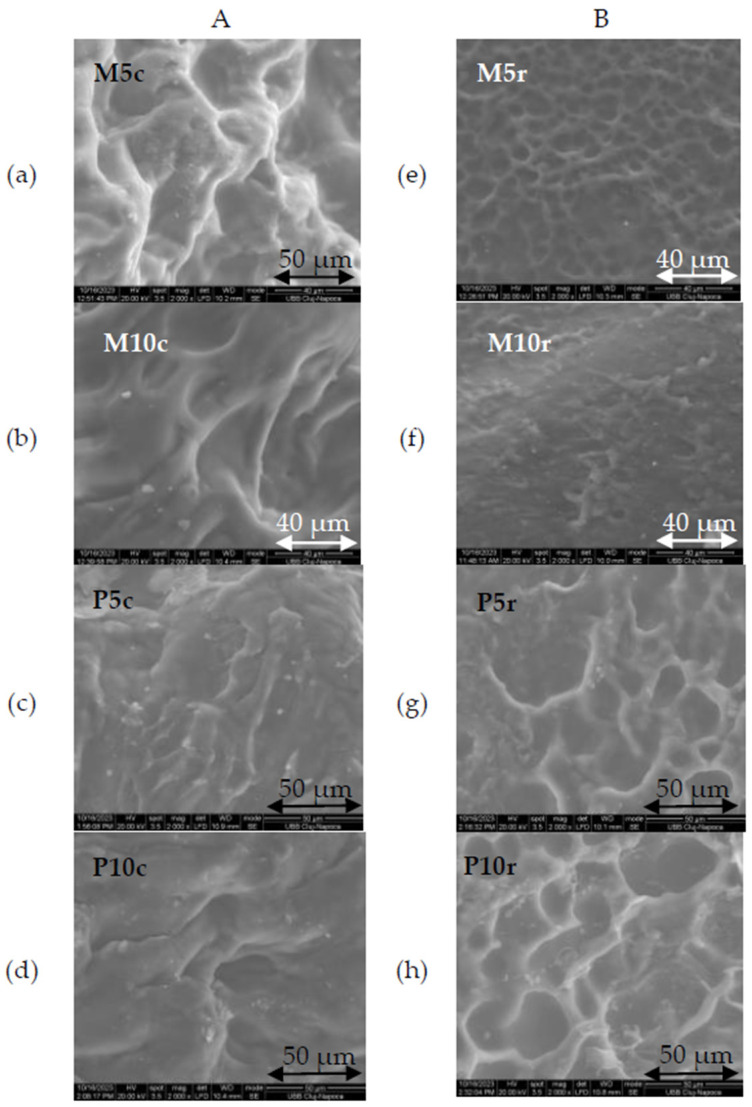
SEM images at 2000× magnification of hydrogels. Column (**A**) includes control samples (**a**) with 5% whey (M5) and (**b**) with 10% whey (M10) along with crosslinked samples (**c**) with 5% whey (P5) and (**d**) with 10% whey (P10) that were frozen before lyophilization. Column (**B**) includes control samples (**e**) with 5% whey (M5) and (**f**) with 10% whey (M10) and crosslinked samples (**g**) with 5% whey (P5) and (**h**) with 10% whey (P10) that were refrigerated before lyophilization.

Scanning Electron Microscopy (SEM) is a valuable tool for analyzing the surface topology and cross-sections of samples, which were prepared by cutting them into small pieces of 5 × 3 mm. In Figure 1A,B, the morphological characteristics of both the control and crosslinked samples are depicted at a magnification of 2000×. 

The variance in the sample composition, coupled with pre-freeze-drying treatment (either refrigeration or freezing), resulted in distinct surface topographies. Notably, the samples characterized by larger pore sizes included the control samples frozen prior to lyophilization, as depicted in Figure 1A(a),(b), as well as those subjected to crosslinking with copper sulfate but refrigerated prior to lyophilization, as shown in Figure 1B(g),(h). Overall, pre-lyophilization refrigeration storage, copper sulfate crosslinking, and a higher concentration of polymeric chains (notably in samples containing 10% whey) enhanced the development of more distinctly defined and denser pore distributions in the hydrogel samples.

Furthermore, the crosslinking facilitated the development of even larger pores, some reaching up to 50 µm in size, as demonstrated in Figure 1A. 

Similar findings were obtained by Kazemi-Taskooh and Varidi [29] for a hydrogel prepared using a different ratio between whey and gellan crosslinked with iron sulfate: the higher the whey and crosslinker concentration, the denser the pore distribution of the hydrogel. The dependence of the surface morphology and porosity on the preparation method has also been reported by Mamidi et al. [30]. They used an acoustic cavity technique to create hydrogels based on zein and carbon nano onions, with superior microstructural, mechanical, biocompatibility, and swelling properties compared to conventional methods. 

Thus, it can be deduced that the interplay of the pre-freeze-drying preparation method, crosslinker presence or absence, and varying concentrations of precursors (in this instance, whey) allows us to tailor specific morphological attributes in hydrogels, which, in turn, confer distinct absorbent properties.

Although lyophilization is quite costly, it unquestionably proves to be a valuable technique in modelling morphological features specific for use in the food industry, transport and controlled release systems, medical engineering, and the environmental sectors.

One can conclude that refrigeration of the samples before freeze-drying leads to the formation of smaller and denser pores in both the control and the copper crosslinked samples.

### 2.2. FTIR Spectroscopy

Changes in the molecular structure of precursors within the hydrogel matrix, induced by gelation, crosslinking, and freeze-drying, can be investigated by FTIR spectroscopy. The complex structure of whey and gelatin proteins, which contain hydrophilic groups such as hydroxyl, amines, and amides, may experience alterations in hydrogen bonding due to the interaction with water molecules. These changes manifest as shifts in the absorption bands of amides, as can be seen in Table 1 and Table 2. 

In the case of Amide A, the absorption bands of the control and crosslinked samples shifted from approximately 3272 cm^−1^, corresponding to the whey and gelatin protein wavelengths obtained in the authors’ previous work [21], to higher wavelengths ranging from 3278.76 cm^−1^ to 3284.5 cm^−1^ for samples refrigerated before freeze-drying, respectively, and from 3280.68 cm^−1^ to 3288.65 cm^−1^ for samples frozen before freeze-drying. Moreover, frozen treatment before lyophilization induced increased intensity of the Amide A bands in the case of samples containing copper compared to those without copper. Those important changes can be correlated to changes in the secondary structure of the protein chains, being affected by hydrogen and van der Waals bonds involved in the formation of helical structures. In our previous work, we showed that the crosslinking process prevented the renaturation of the protein samples, resulting in more disordered structures than in the case of non-crosslinked ones [21].

**Figure 2:**
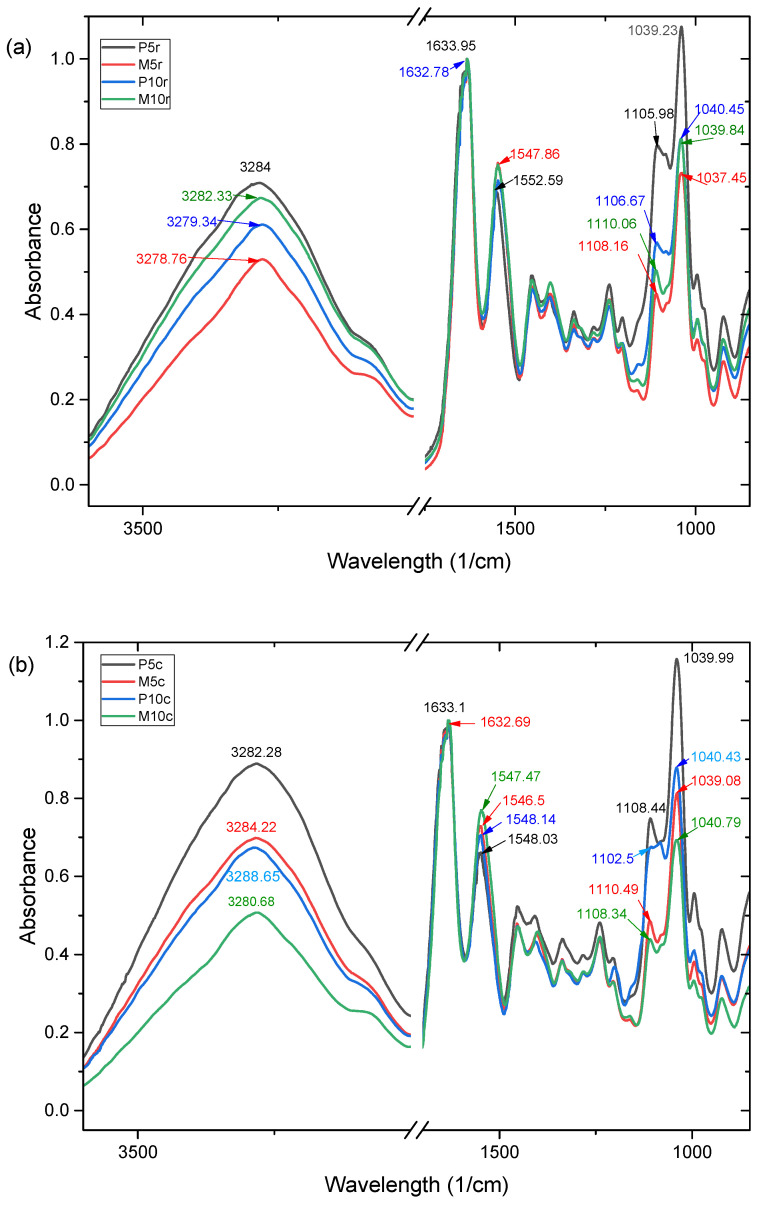
FTIR spectra of hydrogels based on whey and gelatin (M) and crosslinked with copper sulfate (P): (**a**) refrigerated (M5r, M10r, P5r, P10r) prior lyophilization and (**b**) frozen (M5c, M10c, P5c, P10c) prior to lyophilization.

The preparation method of the hydrogels before freeze-drying demonstrated a significant impact on the intensity and wavelengths of the absorption bands. The samples that were frozen before freeze-drying exhibited more intense peaks at high wavenumbers (around 3280 cm^−1^) compared to the refrigerated ones. This phenomenon can be elucidated by considering the influence of the liquid state and solid state of water right before the freeze-drying process on the structure of the hydrogel matrix. Typically, during the freezing process, water undergoes an expansion in volume, leading to disruption of the bonds within the polymer chains, therefore modifying the environment around the amide groups. In contrast, the slower cooling process in refrigerated samples has a less significant impact on the ordered structure of the hydrogel matrix, leading to lower Amide A band intensities, as can be seen in Figure 2.

A significant component of hydrogels is water, given the notable hydration capacity of proteins. Thus, after gelation and crosslinking, the FTIR spectra of the control samples and those crosslinked with copper sulfate exhibit peaks of increased intensity and at wavelengths that differ from those of the precursors, reflecting the interactions between the proteins and remaining water and between the proteins chains. 

On another hand, copper ions from the crosslinker (copper sulfate) can engage in ionic interactions with the functional groups of the polymeric chains (COO^−^), thereby modifying their vibrations and causing significant shifts in the absorption bands of Amide I, from 1630.62 cm^−1^ for gelatin and 1631.72 cm^−1^ for whey, to higher wavelengths around 1632 cm^−1^ and 1633 cm^−1^. Furthermore, the gelation process leads to the formation of new and stronger C=O· · ·H–O hydrogen bonds due to the disruption of C=O· · ·H–N hydrogen bonds in the proteins and the formation of new and stronger C=O· · ·H–O hydrogen bonds between the proteins and glycerol [36]. The addition of copper manifests in visible shifts in the Amide II absorption bands from wavelengths of 1527.47 cm^−1^ for gelatin and 1515.9 cm^−1^ for whey to higher wavelengths in the range of 1547.47 cm^−1^ to 1551.39 cm^−1^ for freeze-dried (see Figure 2b) hydrogels and 1547.45 cm^−1^ and 1552.59 cm^−1^ for refrigerated hydrogels (see Figure 2a). These shifts indicate changes in the molecular interactions that lead to shifts in the vibrational bands and changes in the intensities due to changes in the alpha-helical or beta-sheet content of the proteins.

The C-O stretching in the vibrational bands of the carbonyl group (C=O), the C-N and N-H bonds in the Amide III region of the IR spectrum, shows shifts to higher wavelengths for the hydrogels into the range of 1102 cm^−1^ to 1100 cm^−1^ with respect to the precursors due to the interaction between the proteins, crosslinker, and plasticizers—water and glycerol. 

### 2.3. 1 H NMR Relaxometry

**Figure 3:**
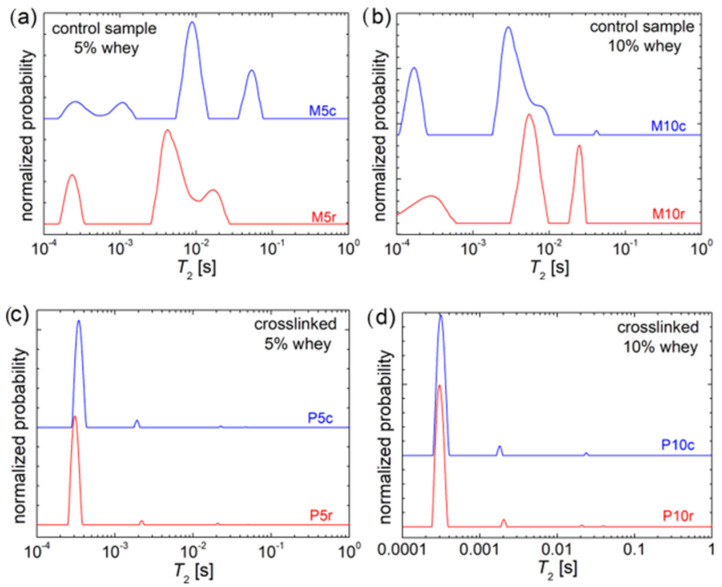
*T*_2_ distributions of refrigerated (r—bottom) and frozen (c—top) hydrogels: (**a**) control samples with 5% whey (M5); (**b**) control samples with 10% whey (M10); (**c**) crosslinked with CuSO_4_ × 5H_2_O and 5% whey (P5); and (**d**) crosslinked with CuSO_4_ × 5H_2_O and 10% whey (P10).

The molecular dynamics are largely influenced by the preparation and storage conditions. ^1^H NMR relaxometry, in particular the distribution transverse relaxation time, *T*_2_, can provide important information about the chain dynamics of produced hydrogels [37]. In Figure 3, the *T*_2_—distributions measured for the refrigerated samples (red curves on bottom) are compared with those measured for the frozen hydrogels (blue curves on top). The effect of the storage temperature (refrigerated at 4 °C or frozen at −18 °C) is evident for the control samples with 5% whey (see Figure 3a) and 10% whey (see Figure 3b). The *T*_2_—distributions present a series of four (only three visible for the M5r sample) peaks located along a wide range. For the control sample with 5% whey, the refrigeration process leads to three distinct components (^1^H pools or reservoirs), which, compared to the sample subjected to the frozen process, are characterized by reduced polymer chain dynamics, as can be seen from the *T*_2_—values, which are lower for the M5r sample than for the M5c sample. One can observe a peak located at *T*_2_—values lower than 1 ms (*T*_2_ ≅ 0.23 ms for M5r and *T*_2_ ≅ 0.26 ms for M5c) characterizing the most rigid components. The frozen sample, M5c, presents a peak with the smallest integral area located at *T*_2_ ≅ 1.07 ms, which can be associated with semi-rigid components. Then, resulting from a numerical deconvolution procedure of the measured *T*_2_—distribution for the M5r refrigerated hydrogel, a doublet of two peaks located at *T*_2_ ≅ 3.92 ms and *T*_2_ ≅ 5.63 ms appears as a single, large and asymmetrical peak, which can be associated with the polymer network characterized by medium dynamics (chain mobility). The corresponding peak for the M5c frozen hydrogel is located at *T*_2_ ≅ 8.84 ms, which indicates increased polymer chain dynamics. The most mobile components corresponding to the M5r refrigerated control hydrogel with 5% whey present a peak in the *T*_2_—distribution located at *T*_2_ ≅ 15.82 ms, while the control hydrogel stored in frozen conditions presents a peak in the *T*_2_—distribution located at *T*_2_ ≅ 53.1 ms, which is significantly larger. Compared to other hydrogels, such as hydrophobically-modified PEG hydrogels for drug delivery [38], where a large amount of hydrogen is characterized by a large relaxation time (large mobility associated with quasi-free water) by low-temperature treatment, the 1H mobility is substantially reduced.

The effect of the increased whey content (from 5% to 10%), especially on the frozen control hydrogel, is significant (see Figure 3b). Therefore, one can globally characterize the M10c (the frozen hydrogel) sample to contain components with reduced polymeric network mobility compared to the M10r sample (the refrigerated hydrogel). Thus, in comparing the blue (top) *T*_2_—distributions from Figure 3a,b, one can observe that 10% of whey leads to (i) a single rigid component (instead of rigid and semi-rigid components) characterized by a *T*_2_ ≅ 0.17 ms; (ii) three semi-mobile components with *T*_2_ ≅ 2.67 ms, 3.76 ms, and 7.70 ms, and (iii) a single peak with a very small integral area (which is proportional to the number of ^1^H in a reservoir) located at *T_2_* ≅ 42.1 ms.

The effect of an increased whey content (from 5% to 10%) on the refrigerated control hydrogel is less significant. One can observe, by comparing the red (bottom) *T*_2_ distributions from Figure 3a,b, that the peak located at the smallest *T*_2_ value (*T*_2_ ≅ 0.28 ms) becomes wider with an increase in the whey content, indicating that the most rigid polymer chains become more heterogeneous and are slightly more mobile. The polymer chains described by medium mobility merge even better in the case of the refrigerated control hydrogel with 10% whey, and one can observe the main peak formed from a doublet of two peaks (observed only after numerical deconvolution) located at *T*_2_ ≅ 5.24 ms and *T*_2_ ≅ 7.07 ms. These values are slightly larger compared to those obtained from the refrigerated control sample with only 5% whey, indicating that an increased amount of whey leads to a slight increase in polymer chain mobility. This effect is more dramatic if one compares the most mobile components; therefore, the M5r sample presents a peak in the *T*_2_—distribution located at *T*_2_ ≅ 15.82 ms, which is shifted to *T*_2_ ≅ 25.00 ms. Moreover, the integral area for this peak increases, also indicating an increase in mobile polymer chains.

From the polymer chain dynamics point of view, the crosslinking process with copper sulfate of whey and gelatin-based hydrogels is crucial, as can be seen in Figure 3c,d. As shown previously by Lopes et al. [21], CuSO_4_ dramatically reduces the polymer chain mobility. The measured *T*_2_ distributions for all four samples (with 5% and 10% of whey and then refrigerated and frozen) are similar. One can observe that the majority of ^1^H is located in the rigid components, as indicated by the presence of a unique narrow peak located at *T*_2_ values of 0.31 ms (P5r), 0.34 ms (P5c), 0.30 ms (P10r), and 0.31 ms (P10r). From this series, one can observe that the frozen process in the crosslinked hydrogel leads to a slight increase in the mobility of the rigid components compared to the refrigerated crosslinked hydrogel. At the same time, the increase in the amount of whey results in a slight decrease in the mobility of the most rigid polymeric chains. Moreover, one can observe a sequence of minor peaks corresponding to small fractions of polymer chains with increased mobility. These are located at (i) *T*_2_ ≅ 2.2 ms, 20.6 ms, and 50.9 ms for the P5r sample; (ii) *T*_2_ ≅ 1.9 ms, 22.5 ms, and 46.9 ms for the P5c sample; (iii) *T*_2_ ≅ 2.0 ms, 20.8 ms, and 39.1 ms for the P10r sample, and (iv) *T*_2_ ≅ 1.8 ms and 23. ms for the P5c sample. The integral area of these peaks diminishes as the *T*_2_ values increase, indicating that the smaller parts of the crosslinked hydrogel exhibit increased mobility at a molecular (polymer chain network) level. Nevertheless, among all the ^1^H NMR relaxometry studies of various types of hydrogels (microgels) [38,39,40], these samples present the most reduced mobility polymer network.

### 2.4. Mechanical Properties

Protein-based hydrogels are known to have mechanical properties that need improvement [41]. 

In this study, whey and gelatin-based hydrogels were reinforced by crosslinking with copper sulfate. Compared to the hydrogels obtained in a previous study [21], which were dried at room temperature, the freeze-dried hydrogels in this study showed a more robust, firmer consistency and texture; therefore, tensile strength and elongation are important attributes for mechanical property investigation. 

**Figure 4:**
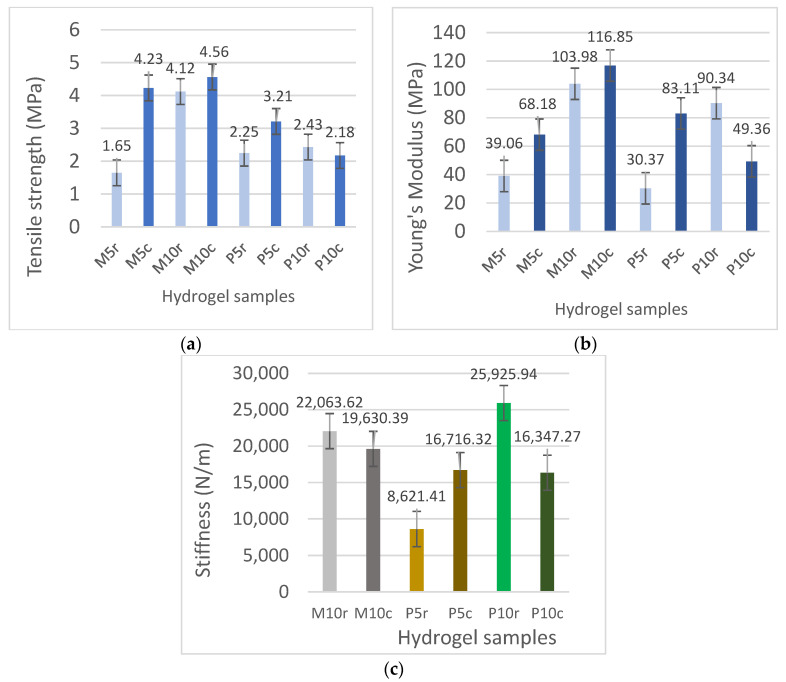
(**a**) Tensile strength (MPa), (**b**) Young’s modulus (MPa), and (**c**) Stiffness (N/m) of freeze-dried hydrogels based on gelatin and whey at concentrations of 5% and 10% (M5, M10) and copper sulfate crosslinked samples (P5, P10), which were refrigerated (r) or frozen (**c**) before lyophilization.

The increase in the whey concentration from 5% to 10% (Figure 4a) led to an increase in the tensile strength from 1.65 MPa (sample M5r) to 4.12 MPa (sample M10r) for the samples refrigerated before freeze-drying and from 4.23 MPa (sample M5c) to 5.56 MPa (sample M10c) for the frozen samples. Increasing the whey concentration led to a denser network within the hydrogel structure, primarily through enhanced hydrogen bonding among the hydrophilic functional groups of the polymeric chains and van der Waals forces. This augmentation in the protein content fosters a more interconnected matrix, attributing to the observed mechanical robustness.

For the crosslinked samples, the increased protein concentration led to a slight increase in the tensile strength of the samples refrigerated prior to freeze-drying (from 2.25 MPa for sample P5r to 2.43 MPa for sample P10r). It seems that ionic bonds involving Cu(II) ions induce significant structural changes in the hydrogels, leading to a decrease in the tensile strength. A low degree of crosslinking allowed for a certain degree of chain slippage, akin to the behavior of elastomers, which resulted in the tensile strength being maintained at values close to those of the non-crosslinked samples containing both 5% WPI (P5r) and 10% WPI (P10r) for the hydrogels refrigerated before lyophilization. It can be concluded that the lyophilization process for the refrigerated samples did not induce major changes in the protein chain interactions. The freezing process significantly increased the tensile strength for the non-crosslinked samples (M5c, M10c), a property less dependent on the whey concentration. Consequently, the structures resulting from freezing became less elastic. Copper crosslinking of the frozen samples led to a decrease in the tensile strength due to the ionic bonds formed between the protein chains through divalent copper ions, indicating a low degree of crosslinking, which conferred desirable properties to the hydrogels. An increase in the whey concentration to 10% led to a decrease in the tensile strength from 3.21 MPa (sample P5c) to 2.18 MPa (sample P10c), illustrating the profound changes induced by the freezing process to the morphology of the lyophilized hydrogels. The investigation into the effects of the protein concentration and copper sulfate crosslinking on the mechanical properties of the hydrogels revealed several interesting findings. The anticipated trend of increased tensile strength and Young’s modulus with higher protein concentrations held true for both the refrigerated and frozen stored hydrogels before lyophilization due to the strong interactions between the protein chains involving both the hydrogen bonds between the hydrophilic functional groups and van der Waals interactions. Copper sulfate crosslinking enhanced the tensile strength only in the 5% whey protein samples, while in the 10% whey protein samples, it led to a decrease in the tensile strength for previously frozen samples. 

Based on the data from Figure 4b regarding Young’s modulus and stiffness (see Figure 4c), a concise interpretation would emphasize the correlation between the hydrogel composition, processing conditions, and mechanical properties. For Young’s modulus, an upward trend with increasing protein concentration suggests a more rigid network structure, potentially due to denser intermolecular interactions. Meanwhile, variations in the stiffness indicate how the hydrogel’s response to mechanical stress is influenced by both the degree of crosslinking and the pre-lyophilization treatment (refrigerated vs. frozen), with temperature treatments affecting the polymer network’s integrity and elasticity. These insights are crucial for tailoring hydrogel properties for specific applications by manipulating the protein content and processing conditions.

The varied composition of hydrogel formulations based on whey leads to distinct interactions within the hydrogel matrix, manifesting unique behaviors for each hydrogel type. This observation is further supported by another study [41], wherein Zhang et al. documented an escalation in tensile strength values corresponding to the whey protein isolate:psyllium seed gum ratio, which progressed from 1:0 to 3:1 and reached a maximum at 1:1. Furthermore, gelatin is a commonly used precursor in the formulation of hydrogels and contributes to an improvement in their mechanical properties, as also reported in other studies [42].

## 3. Conclusions

This study highlights the significant impact of thermal pretreatment methods on the properties of hydrogel samples before lyophilization. When comparing hydrogels subjected to freezing before lyophilization, SEM images reveal larger pores with thicker walls, contributing to a more robust matrix structure which results in an observable increase in the tensile strength after lyophilization. On the other hand, hydrogels subjected to refrigeration show FTIR absorption bands indicating changes in the secondary structure of proteins. Stiffness variations show that refrigeration can influence mechanical properties, with a doubled whey concentration leading to decreased stiffness and a minimal whey concentration resulting in increased stiffness. Furthermore, the crosslinking process significantly diminishes the impact of the whey content and storage conditions, establishing a polymeric chain network characterized by reduced mobility in all the samples. In summary, the method of thermal pretreatment, whether freezing or refrigerating before lyophilization, distinctly shapes the characteristics of hydrogels, offering insights for tailoring formulations to specific application requirements.

## 4. Materials and Methods

### 4.1. Materials and Hydrogel Preparation Method

The hydrogels were obtained from Whey Protein Isolate (WPI) ISOLAC produced by the Carbery Group Carbery (Cork, Ireland) and distributed by S.C. Way Better Nutrition from Cluj-Napoca, the gelatin reagent was obtained from AMRESCO LLC (Fountain Parkway Solon, Cleveland, OH, USA) and glycerol was obtained from Sigma-Aldrich (Taufkirchen, Germany). The crosslinker used was copper sulfate pentahydrate (CuSO_4_ × 5H_2_O) obtained from Sigma-Aldrich, Taufkirchen, Germany. The hydrogels were obtained according to a method described in a previous work [21]. For clarity, two crosslinked samples were formulated using solutions containing 5% and 10% whey (W) (g/g), gelatin (Ge) (g), 50% glycerol (g/g), and 5% CuSO_4_ × 5H_2_O (g/g). Simultaneously, for two additional samples with identical polymer and glycerol compositions, the CuSO_4_ ×5H_2_O solution was substituted with distilled water (DW) (g). The initial set of samples was designated as P5 and P10, while the latter were denoted as M5 and M10. 

Following 24 h of room-temperature drying, the samples were subjected to lyophilization. Before this step, they were cooled, partly by refrigeration at 4 °C (sample r) and partly by freezing at −18 °C (sample c), providing additional insight into this process. The percentage concentration of the precursors at the end of the gelation process in this study is given in Table 3.

### 4.2. Scanning Electron Microscope (SEM) 

The surface of the samples was analyzed microscopically using the Inspect Scanning Electron Microscope (FEI Company, Hillsboro, OR, USA) under low vacuum at room temperature with a 3.5 spot and ×2000 magnification [29,30].

### 4.3. FTIR Spectroscopy

The changes in the amide bands of the whey and gelatin proteins after freeze-drying were investigated using FTIR absorption bands obtained using a Bruker tensor 27 FTIR spectrometer (Bruker Optik GmbH, Ettlingen, Germany) in attenuated total reflection (ATR) mode [22]. 

### 4.4. ^1^H NMR Relaxometry

Spin-spin ^1^H relaxation measurements (*T*_2_) of the hydrogels were performed using a Bruker Minispec mq20 NMR spectrometer (Bruker, Karlsruhe, Germany) by Inverse Laplace Transform of CPMG (Carr-Purcell-Meiboom-Gill) decays. A total of 1000 echoes was recorded with an echo time of 70 µs and a recycle delay of 0.5 s. To ensure an optimal signal-to-noise ratio, 512 scans were conducted [43].

### 4.5. Mechanical Properties

The aim of this investigation was to identify the influence of the storage conditions prior to freeze-drying on the hydrogel composition and the crosslinking effect on the mechanical properties of whey and gelatin-based hydrogels that would allow their use as absorbents for food packaging, packaging films, wound dressings, or tissue reconstruction biomaterials in surgery. The samples underwent stretching at a rate of 100 mm/min, and measurements for the tensile strength, stiffness, and Young’s modulus were obtained using a Lloyd LR5k Plus dual-column mechanical testing machine (Lloyd Instruments, London, UK).

## Figures and Tables

**Table 1:** FTIR absorption bands of whey and gelatin-based hydrogels refrigerated prior to lyophilization.

Gelatin	Whey	M5r	M10r	P5r	P10r	Attributions
(cm^−1^)						
3272.82	3272.4	3278.76	3282.33	3284.06	3279.34	Amide A, tensile vibrations NH [21,31,32], hydrogen bonds [21,22]
1630.62	1631.72	1632.93	1638.96	1633.95	1632.78	Amide I, C=O and NH vibrations [21,31,33], H bonds coupled with COO^−^ [22,34]
1527.47	1515.90	1547.86	1547.78	1552.59	1547.45	Amide II, CN vibrations/stretching, NH bending [21,22,32,35]
1080.02	1076.17	1108.16	1110.06	1105.98	1106.67	Amide III CN and NH vibrations [21,22,35]

**Table 2:** FTIR absorption bands of whey and gelatin-based hydrogels frozen prior to lyophilization.

Gelatin	Whey	M5c	M10c	P5c	P10c	Attributions
(cm^−1^)						
3272.82	3272.4	3284.22	3280.68	3282.28	3288.65	Amide A, tensile vibrations NH [21,31,32], hydrogen bonds [21,22]
1630.62	1631.72	1632.69	1632.57	1633.1	1633.4	Amide I, C=O and NH vibrations [21,31,33] H bonds coupled with COO^−^ [22,34]
1527.47	1515.90	1551.39	1547.47	1548.03	1548.14	Amide II, CN vibrations/stretching, NH bending [21,22,32,35]
1080.02	1076.17	1110.49	1108.34	1108.44	1082.09	Amide III CN and NH vibrations [21,22,35]

**Table 3:** The percentage composition of precursors (whey, gelatin, glycerol, CuSO_4_ × 5H_2_O, and water) in the formulation of 5% and 10% whey hydrogels.

Code Sample	W 5%	W 10%	Gly 1:1	Ge	CuSO_4_ × 5H_2_O 5%	Distilled Water	Total
	(%)						
M5	3.13	0	6.25	7.81	0	82.81	100.00
M10	0	6.25	6.25	7.81	0	79.69	100.00
P5	3.13	0	6.25	7.81	0.86	81.95	100.00
P10	0	6.25	6.25	7.81	0.86	78.83	100.00

## Data Availability

The data presented in this study are openly available in article.
